# Src-homology protein tyrosine phosphatase-1 agonist, SC-43, reduces liver fibrosis

**DOI:** 10.1038/s41598-017-01572-z

**Published:** 2017-05-11

**Authors:** Tung-Hung Su, Chung-Wai Shiau, Ping Jao, Nian-Jie Yang, Wei-Tien Tai, Chun-Jen Liu, Tai-Chung Tseng, Hung-Chih Yang, Chen-Hua Liu, Kai-Wen Huang, Ting-Chen Hu, Yu-Jen Huang, Yao-Ming Wu, Li-Ju Chen, Pei-Jer Chen, Ding-Shinn Chen, Kuen-Feng Chen, Jia-Horng Kao

**Affiliations:** 1 Division of Gastroenterology and Hepatology, Department of Internal Medicine, National Taiwan University Hospital, Taipei, 10002 Taiwan; 2 Hepatitis Research Center, National Taiwan University Hospital, Taipei, 10002 Taiwan; 3 Graduate Institute of Clinical Medicine, National Taiwan University College of Medicine, Taipei, 10002 Taiwan; 4 Institute of Biopharmaceutical Sciences, National Yang-Ming University, Taipei, 11221 Taiwan; 5 National Center of Excellence for Clinical Trial and Research, National Taiwan University Hospital, Taipei, 10002 Taiwan; 6 Department of Internal Medicine, National Taiwan University Hospital Jinshan Branch, New Taipei City, 20844 Taiwan; 7 Department of Surgery, National Taiwan University Hospital, Taipei, 10002 Taiwan; 8 Department of Medical Research, National Taiwan University Hospital, Taipei, 10002 Taiwan; 9 Genomics Research Center, Academia Sinica, Taipei, Nankang 11529 Taiwan

## Abstract

This study aimed to investigate the role of src-homology protein tyrosine phosphatase-1 (SHP-1)–signal transducer and activator of transcription 3 (STAT3) pathway in liver fibrogenesis and the anti-fibrotic effect of SHP-1 agonist. The antifibrotic activity of SC-43, a sorafenib derivative with an enhanced SHP-1 activity, was evaluated in two fibrosis mouse models by carbon tetrachloride induction and bile duct ligation. Rat, human, and primary mouse hepatic stellate cells (HSCs) were used for mechanistic investigations. The results showed that SHP-1 protein primarily localized in fibrotic areas of human and mouse livers. SC-43 treatment reduced the activated HSCs and thus effectively prevented and regressed liver fibrosis in both fibrosis mouse models and improved mouse survival. *In vitro* studies revealed that SC-43 promoted HSC apoptosis, increased the SHP-1 activity and inhibited phospho-STAT3. The enhanced SHP-1 activity in HSCs significantly inhibited HSC proliferation, whereas SHP-1 inhibition rescued SC-43-induced HSC apoptosis. Furthermore, SC-43 interacted with the N-SH2 domain of SHP-1 to enhance the activity of SHP-1 as its antifibrotic mechanism. In conclusion, the SHP-1–STAT3 pathway is crucial in fibrogenesis. SC-43 significantly ameliorates liver fibrosis through SHP-1 upregulation. A SHP-1-targeted antifibrotic therapy may represent a druggable strategy for antifibrotic drug discovery.

## Introduction

Chronic hepatitis caused repeated liver inflammation and gradually evolved into liver cirrhosis and hepatocellular carcinoma (HCC). Patients with chronic hepatitis B (CHB) related liver cirrhosis harbor a 12-fold increased risk of HCC^1^. In addition to HCC, liver cirrhosis is associated with variceal bleeding, spontaneous bacterial peritonitis, hepatic encephalopathy, and hepatorenal syndrome. Liver cirrhosis is a major health issue; however, an effective therapy against liver fibrosis is presently unavailable. An antifibrotic treatment is a critical unmet clinical requirement^2^.

Discovering the genetic regulation of fibrosis and the liver-specific feature of fibrogenesis is crucial for identifying a mechanism-based antifibrotic therapy^3^. The signal transducer and activator of transcription 3 (STAT3) pathway in hepatic stellate cells (HSCs) regulate their survival, proliferation, and activation^4^. Adiponectin was recently reported to have an antifibrotic activity in the liver by inhibiting the Janus kinase (JAK)–STAT pathway^5^. According to the aforementioned studies, STAT3 may play a major role in hepatic fibrogenesis.

**Figure 1:**
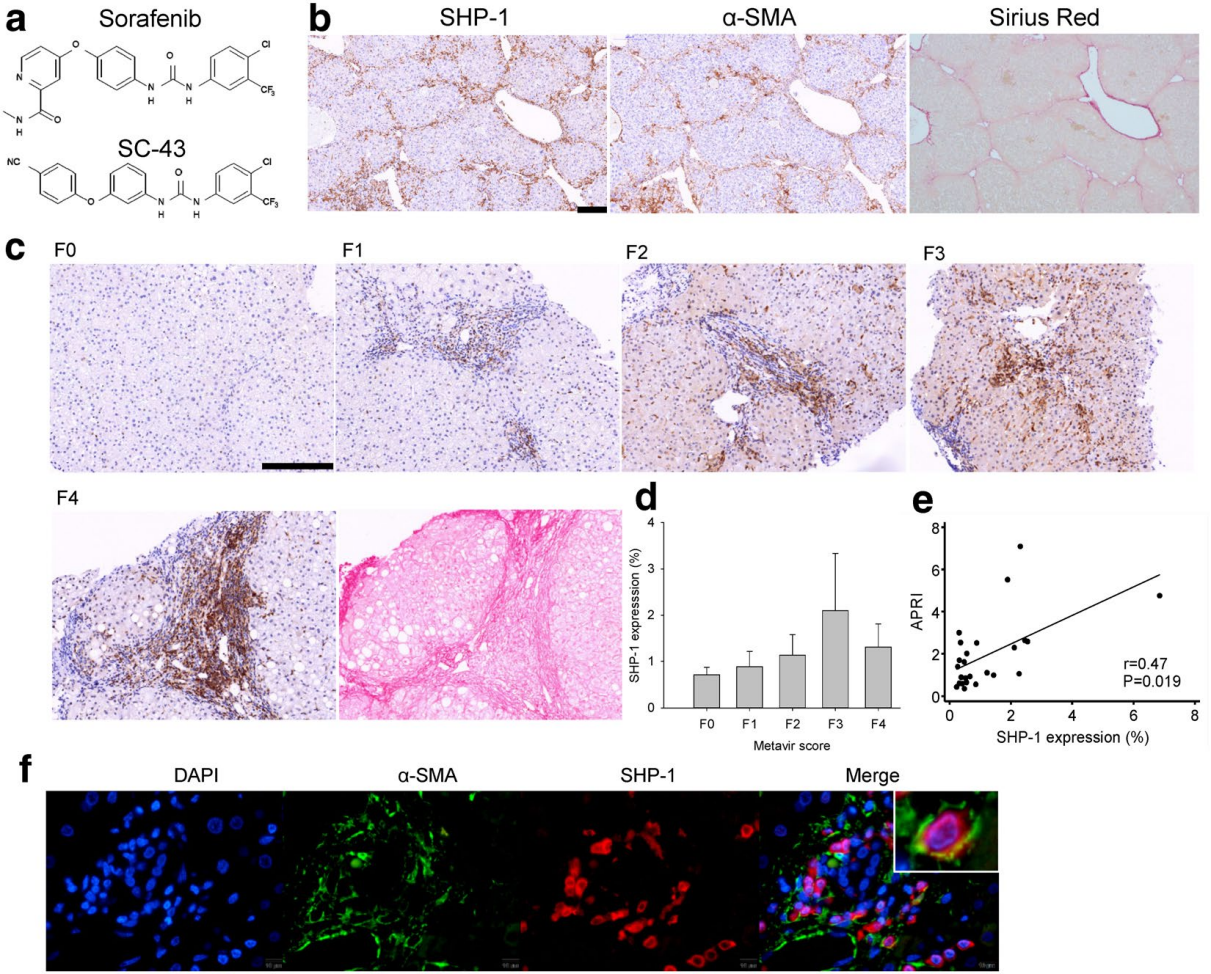
SHP-1 phosphatase is associated with liver fibrosis. (**a**) The structure of sorafenib and its derivative SC-43. (**b**) SHP-1 located primarily in areas with α-SMA-expressed activated HSCs and significant fibrosis by sirius red staining of the CCl_4_-induced fibrosis mouse liver. (**c**) SHP-1 overexpression in fibrotic areas of patients with CHB with advanced fibrosis, graded by the Metavir scores (F0–F4). The SHP-1 located in sirius red-positive fibrotic areas. (**d**) SHP-1 expression according to Metavir score. (**e**) SHP-1 expression positively correlated with APRI. (**f**) SHP-1 colocalized with α-SMA-expressed activated HSCs in human liver. Scale bar: 200 μm.

Our recent data revealed that sorafenib and its derivative, SC-1 (without the Raf kinase inhibition activity)^6^, inhibit the proliferation of HCC cell lines through STAT3 inactivation^7, 8^. Furthermore, both sorafenib and SC-1 ameliorated liver fibrosis *in vivo* and promoted HSC apoptosis *in vitro* through STAT3 pathway inhibition^9^. We confirmed that STAT3 is crucial in fibrogenesis and suggested that STAT3 is a potential target for antifibrotic therapy in patients with CHB related liver fibrosis^9^. Furthermore, a novel sorafenib derivative, SC-43, (Fig. 1a) was developed to display a more potent anti-HCC activity than does sorafenib, as measured by the enhanced Src-homology protein tyrosine phosphatase (SHP)-1 activity, phospho-STAT3 (P-STAT3) inhibition, apoptosis induction, even in sorafenib-resistant HCC cells, and showed more desirable survival benefits than did sorafenib in orthotopic HCCs^10^.

Our hypothesis is that the SHP-1–STAT3 pathway is involved in liver fibrogenesis. In this study, we investigated the role of SHP-1 in fibrogenesis. The antifibrotic activity of the SHP-1 agonist, SC-43, and its antifibrotic mechanism were further explored.

## Results

### SHP-1 phosphatase is associated with liver fibrosis

STAT3 is involved in fibrogenesis; therefore, we first investigated the expression of SHP-1, an inhibitor of P-STAT3, in fibrotic liver. After CCl_4_ induction for 4 weeks, SHP-1 primarily located in areas with α-SMA-expressed activated HSCs and significant fibrosis (Fig. 1b). We further investigated SHP-1 expression in patients with CHB with different degrees of fibrosis. SHP-1 tended to increase in patients with advanced fibrosis (Fig. 1c,d). SHP-1 expression correlated well with APRI (Fig. 1e) and serum ALT level (Supplementary Fig. S1). The SHP-1 colocalized with α-SMA-expressed activated HSCs (Fig. 1f).

Using Sodium stibogluconate to inhibit SHP-1 expression, the degree of fibrosis significantly increased after CCl_4_ induction and tended to increase after BDL compared with the vehicle-treated group (Supplementary Figs S2, S3). These data suggested that SHP-1 is involved in active hepatic fibrogenesis.

### SC-43 treatment ameliorates liver fibrosis in CCl_4_ liver fibrosis mouse models

**Figure 2:**
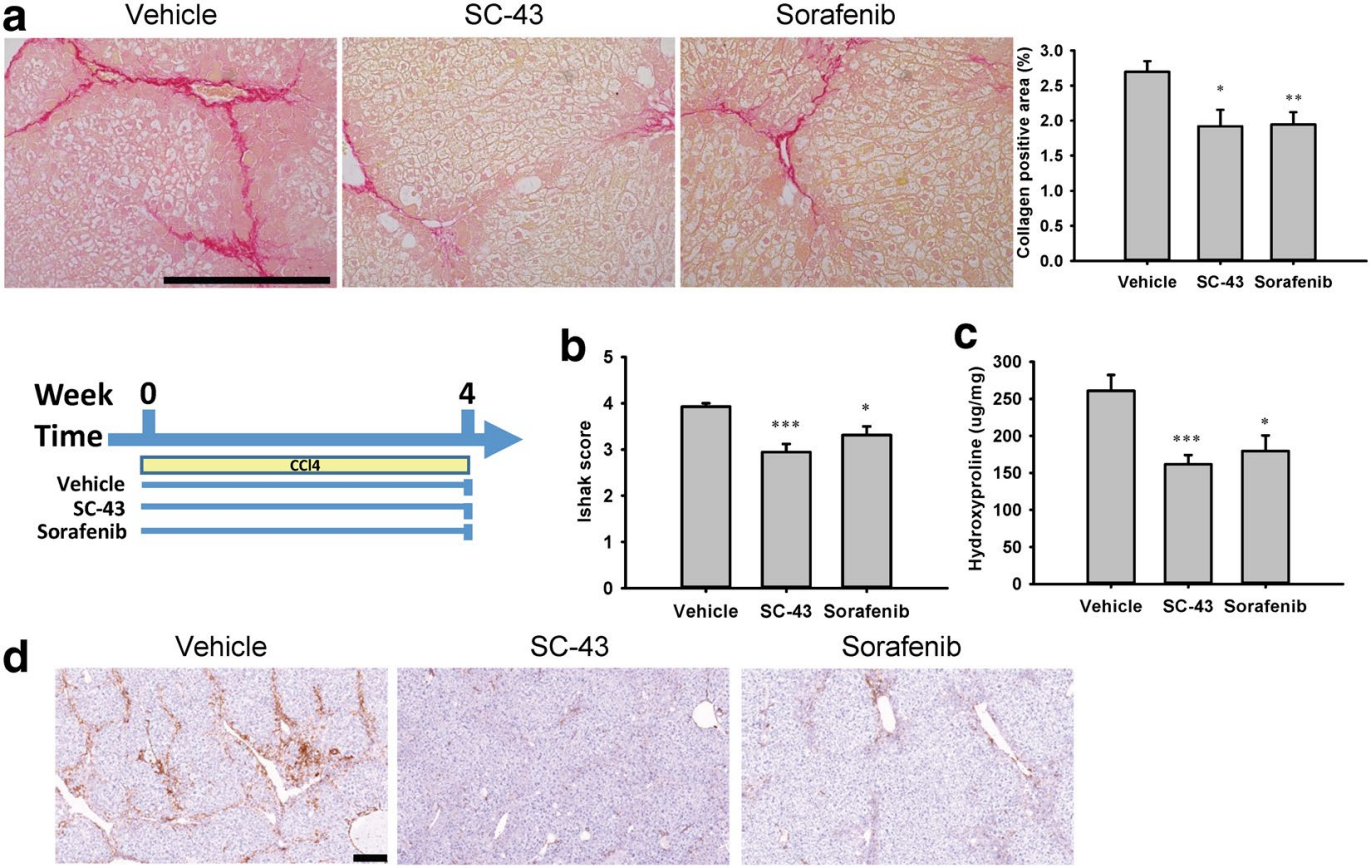
SC-43 treatment ameliorates liver fibrosis in the CCl_4_-induced liver fibrosis mouse prevention model. In the fibrosis prevention model, SC-43 (10 mg/kg) and sorafenib (10 mg/kg) were concurrently administered with CCl_4_ for 4 weeks, and significant fibrosis regression was observed. (**a**) Representative images after sirius red staining with the collagen positive area quantification. (**b**) Ishak fibrosis score. (**c**) Liver hydroxyproline quantification. (**d**) α-SMA staining. Scale bar: 200 μm. No significant difference between SC-43 and sorafenib group. Columns: mean and bars: standard error. *P < 0.05, **P < 0.01, and ***P < 0.001 compared with the vehicle group. N = 7–9 in each group.

SC-43 treatment increases greater SHP-1 activity than sorafenib does. Therefore, we hypothesized that SC-43 has a more favorable antifibrotic activity than does sorafenib. In the fibrosis prevention model, SC-43 and sorafenib were concurrently administered with CCl_4_ for 4 weeks. Following the SC-43 and sorafenib treatment, significant fibrosis regression was observed through sirius red staining, the collagen-positive area, the Ishak fibrosis score, the hydroxyproline level, and α-SMA expression in liver tissues (Fig. 2). Compared with the vehicle-treated group, the positive area of α-SMA tended to reduce (Supplementary Fig. S4) and apoptotic HSCs were significantly increased after SC-43 treatment (Supplementary Fig. S5).

**Figure 3:**
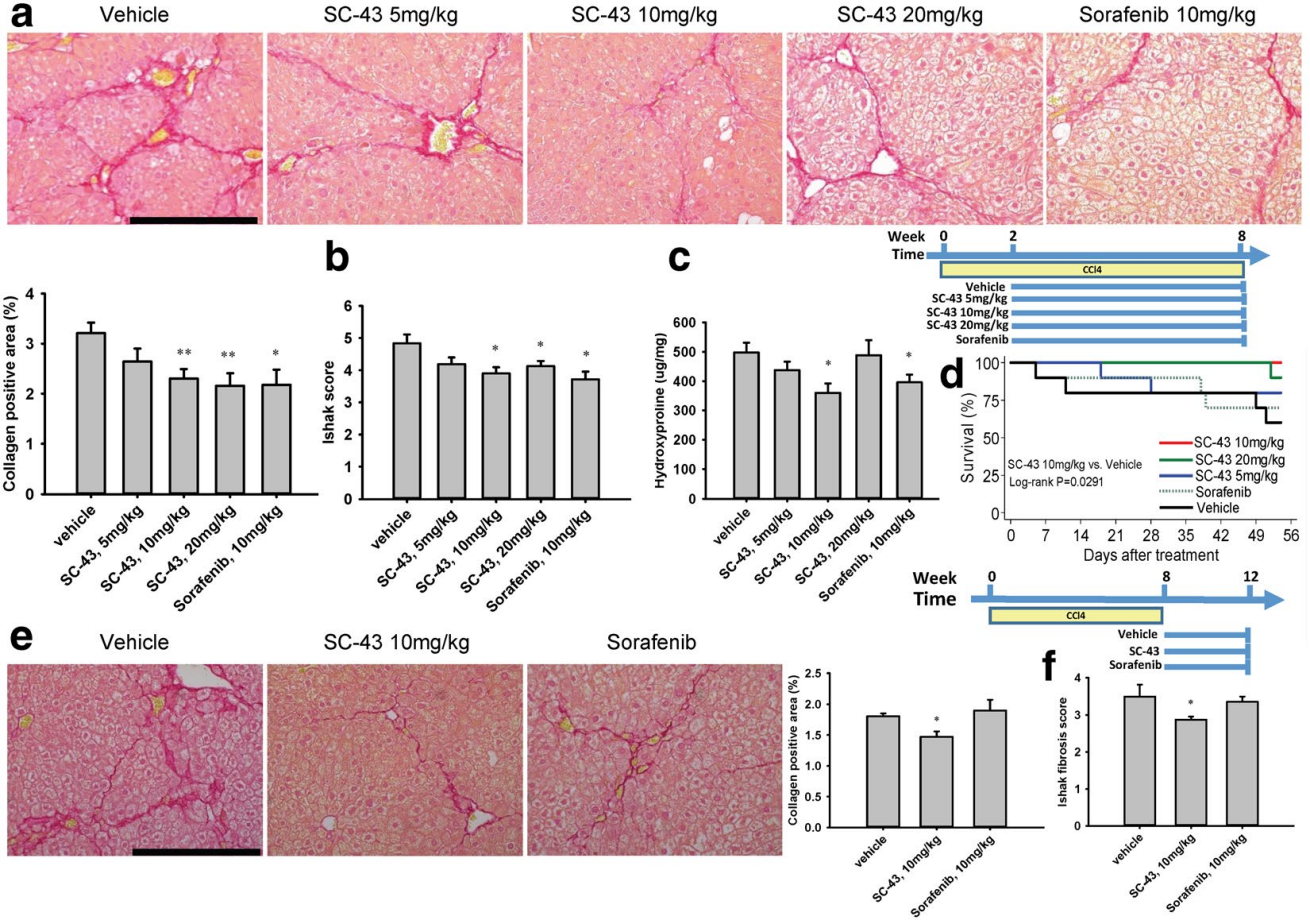
SC-43 treatment ameliorates liver fibrosis in the CCl_4_-induced liver fibrosis mouse treatment and regression model. In the fibrosis treatment model, following CCl_4_ induction for 2 weeks, SC-43 at various dosages and sorafenib (10 mg/kg) were concurrently administered with CCl_4_ for the subsequent 6 weeks. Fibrosis improved significantly after the SC-43 and sorafenib treatment. (**a**) Representative image after sirius red staining and the collagen-positive area quantification. (**b**) Ishak fibrosis score. (**c**) Liver hydroxyproline quantification. (**d**) The SC-43 (10  mg/kg) treatment improved mouse survival compared with the vehicle group (log-rank P = 0.0291). N = 6–10 in each group. In the fibrosis regression model, after CCl_4_ induction for 8 weeks, SC-43 (10  mg/kg) and sorafenib (10  mg/kg) were administered for the following 4 weeks without CCl_4_. Fibrosis regressed significantly after the SC-43 treatment. (**e**) Representative image after sirius red staining and the collagen-positive area quantification. (**f**) Ishak fibrosis score. N = 6–8 in each group. Scale bar: 200 μm. Columns: mean and bars: standard error. *P < 0.05 and **P < 0.01 compared with the vehicle group.

In the fibrosis treatment model, mild liver fibrosis (Ishak score, 2–3) was achieved after CCl_4_ induction for 2 weeks. SC-43 (5, 10, or 20 mg/kg) and sorafenib (10 mg/kg) were concurrently administered with CCl_4_ in the following 6 weeks. The SC-43 and sorafenib treatment significantly ameliorated liver fibrosis, as observed through sirius red staining (Fig. 3a). A dose–response trend of an increasing antifibrotic activity of SC-43 was observed in the collagen-positive area, Ishak fibrosis score, and hydroxyproline level (Fig. 3a–c). Furthermore, the SC-43 (10 mg/kg) treatment significantly improved the survival compared with the control group (log-rank P = 0.0291) and a tendency to yield higher survival than does the sorafenib treatment (log- rank P = 0.0671; Fig. 3d). We chose SC-43 10 mg/kg for subsequent experiments.

In the fibrosis regression model, advanced fibrosis and cirrhosis (Ishak score, 4–6) developed through CCl_4_ induction for 8 weeks. SC-43 (10 mg/kg) and sorafenib (10 mg/kg) were administered for the following 4 weeks without CCl_4_. At sacrifice, we observed that hepatic fibrosis regressed even in the control group (Fig. 3e, compared with Fig. 3a), indicating spontaneous fibrosis regression after discontinuing CCl_4_ induction for 8 weeks. However, we observed a significantly decreased collagen-positive area and Ishak fibrosis score in the SC-43 treatment group (Fig. 3e,f).

### SC-43 treatment ameliorates liver fibrosis in bile duct ligation liver fibrosis mouse models

**Figure 4:**
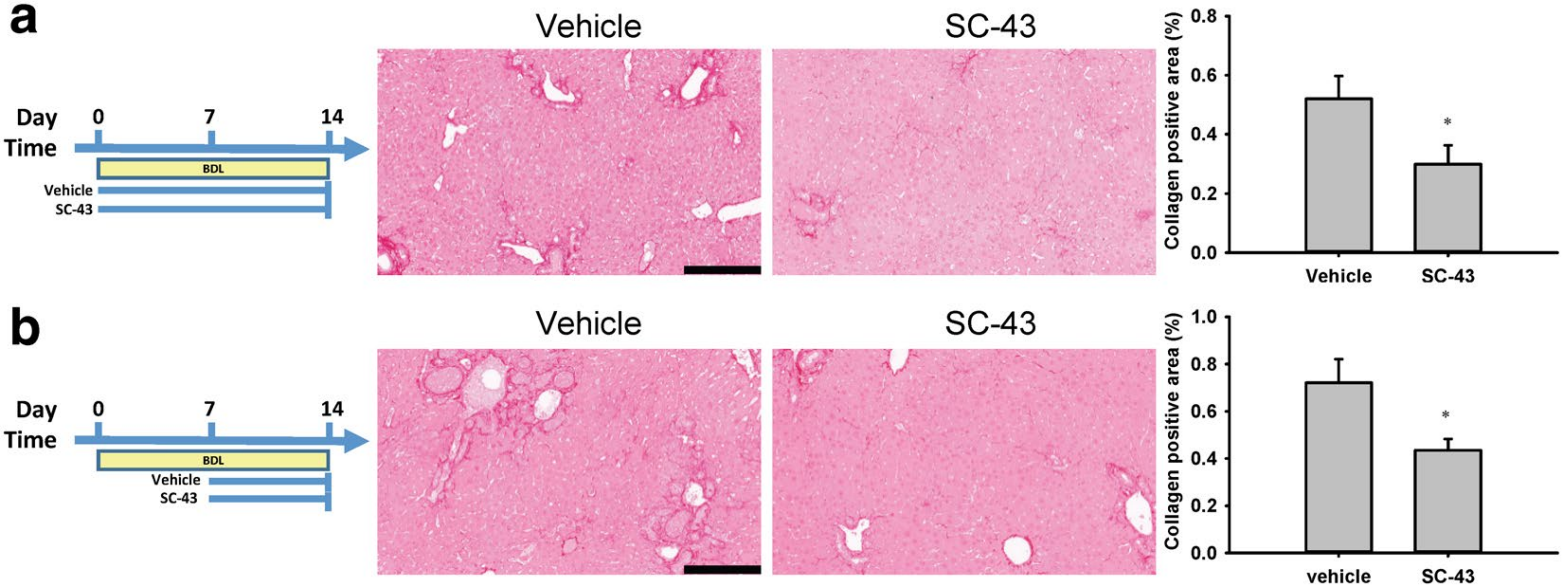
SC-43 treatment ameliorates liver fibrosis in the bile duct ligation liver fibrosis mouse model. In the BDL fibrosis prevention model, the vehicle and SC-43 (10  mg/kg) were administered from day 1 until sacrifice on day 14 of BDL. SC-43 treatment yielded significant fibrosis regression. (**a**) Representative images after sirius red staining and the collagen-positive area quantification. In the BDL fibrosis therapy model, the vehicle and SC-43 (10  mg/kg) were administered since day 8 until sacrifice on day 14 after BDL. The SC-43 treatment yielded significant fibrosis regression. (**b**) Representative images after sirius red staining and the collagen-positive area quantification. Scale bar: 200 μm. Columns: mean and bars: standard error. *P < 0.05 compared with the vehicle group. N = 7–8 in each group.

We further investigated the antifibrotic activity of SC-43 in the BDL model. In the fibrosis prevention model, the vehicle and SC-43 (10 mg/kg) were administered from day 1 until sacrifice on day 14 of BDL. The SC-43 treatment significantly reduced the collagen-positive area (Fig. 4a). In the fibrosis treatment model, the vehicle and SC-43 (10 mg/kg) were administered from day 8 until sacrifice on day 14 following BDL. The SC-43 treatment also significantly reduced the collagen-positive area (Fig. 4b). The hydroxyproline level slightly reduced after SC-43 treatment in both the prevention and treatment models (Supplementary Fig. S6).

These animal study results suggested the antifibrotic activity of SC-43 in the prevention, treatment, and regression of liver fibrosis.

### SC-43 induces the apoptosis of hepatic stellate cells through signal transducer and activator of transcription 3 inhibition

**Figure 5:**
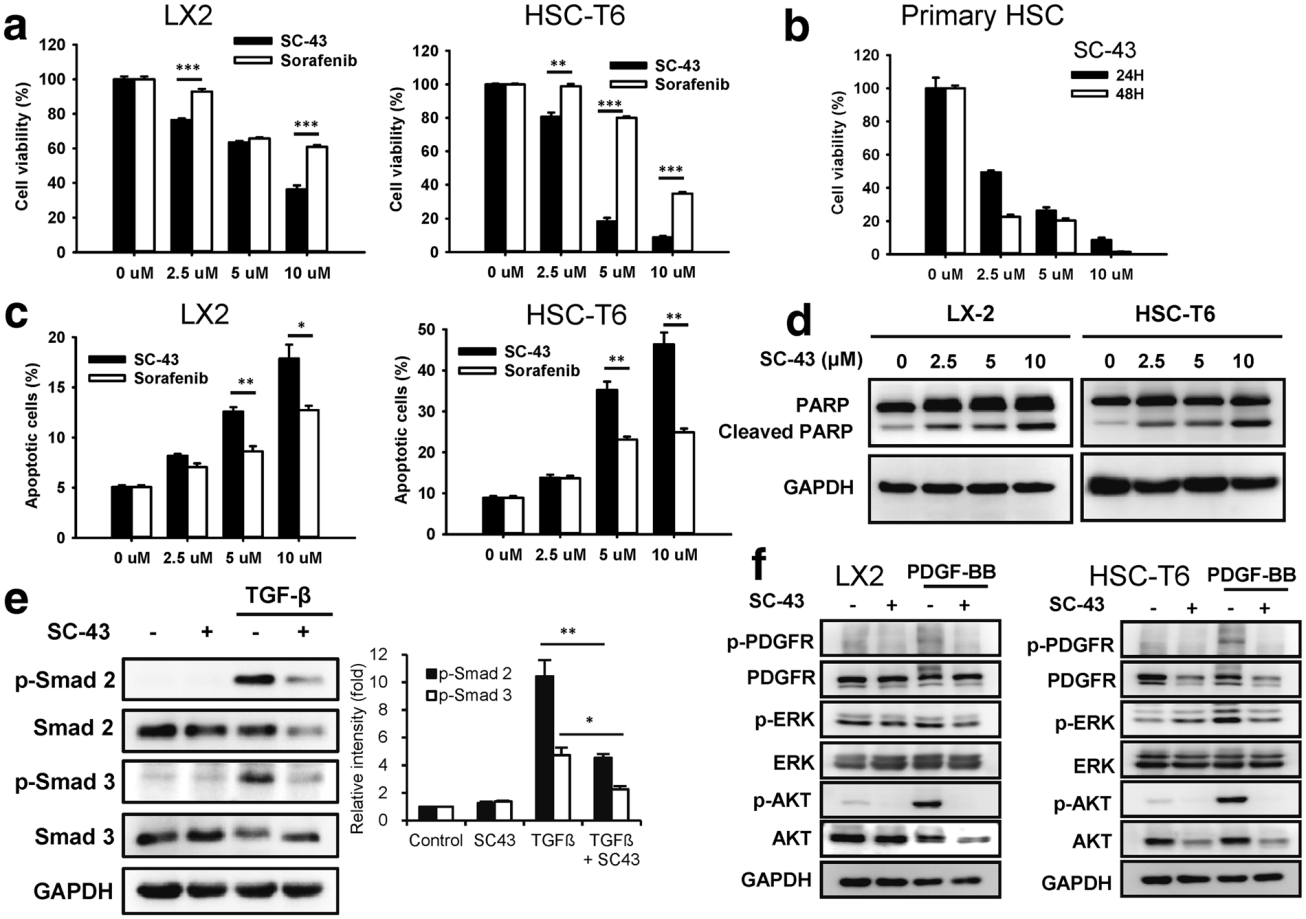
SC-43 treatment induces apoptosis of hepatic stellate cells. (**a**) Dose-escalation effects of SC-43 and sorafenib administered for 24 h on the cell viability of LX2 and HSC-T6 cells. (**b**) Dose-escalation and time-dependent effects of SC-43 administration for 24 h or 48 h on the cell viability of primary mouse HSCs. (**c**) Dose-escalation effects of SC-43 and sorafenib administration for 24 h on the apoptosis of LX2 and HSC-T6 cells. (**d**) SC-43 induced LX2 and HSC-T6 apoptosis by increasing the cleaved PARP fragments. (**e**) The SC-43-induced downregulation of the TGF-β pathway in LX2 cells. (**f**) The SC-43-induced downregulation of p-PDGFR and p-Akt in the PDGF pathway in LX2 and HSC-T6 cells. Columns: mean and bars: standard error (n = 3–4). *P < 0.05, **P < 0.01, and ***P < 0.001.

The antifibrotic mechanism of SC-43 was further investigated *in vitro*. The SC-43 treatment had dose-dependent effects to reduce the viability of HSC-T6 and LX2 cells, more significant than sorafenib (Fig. 5a). The SC-43 treatment also exerted dose- and time-dependent effects to reduce the viability of primary mouse HSCs (Fig. 5b). In addition, compared with sorafenib, SC-43 significantly increased HSC apoptosis in a dose-dependent manner (Fig. 5c). SC-43 treatment significantly increased the Sub-G1 phase of cell cycles (Supplementary Fig. S7). Western blotting revealed that SC-43 dose-dependently increased the cleavage of PARP fragments (Fig. 5d).

TGF-β and PDGFR are major canonical pathways involved in fibrogenesis and HSC activation and proliferation. We first investigated the effects of SC-43 on TGF-β and PDGFR pathways. SC-43 significantly downregulated p-Smad2 and p-Smad3 of the TGF-β pathway in LX2 cells (Fig. 5e). It also downregulated p-PDGFR and p-Akt in the PDGFR pathway both in HSC-T6 and LX2 cells (Fig. 5f).

**Figure 6:**
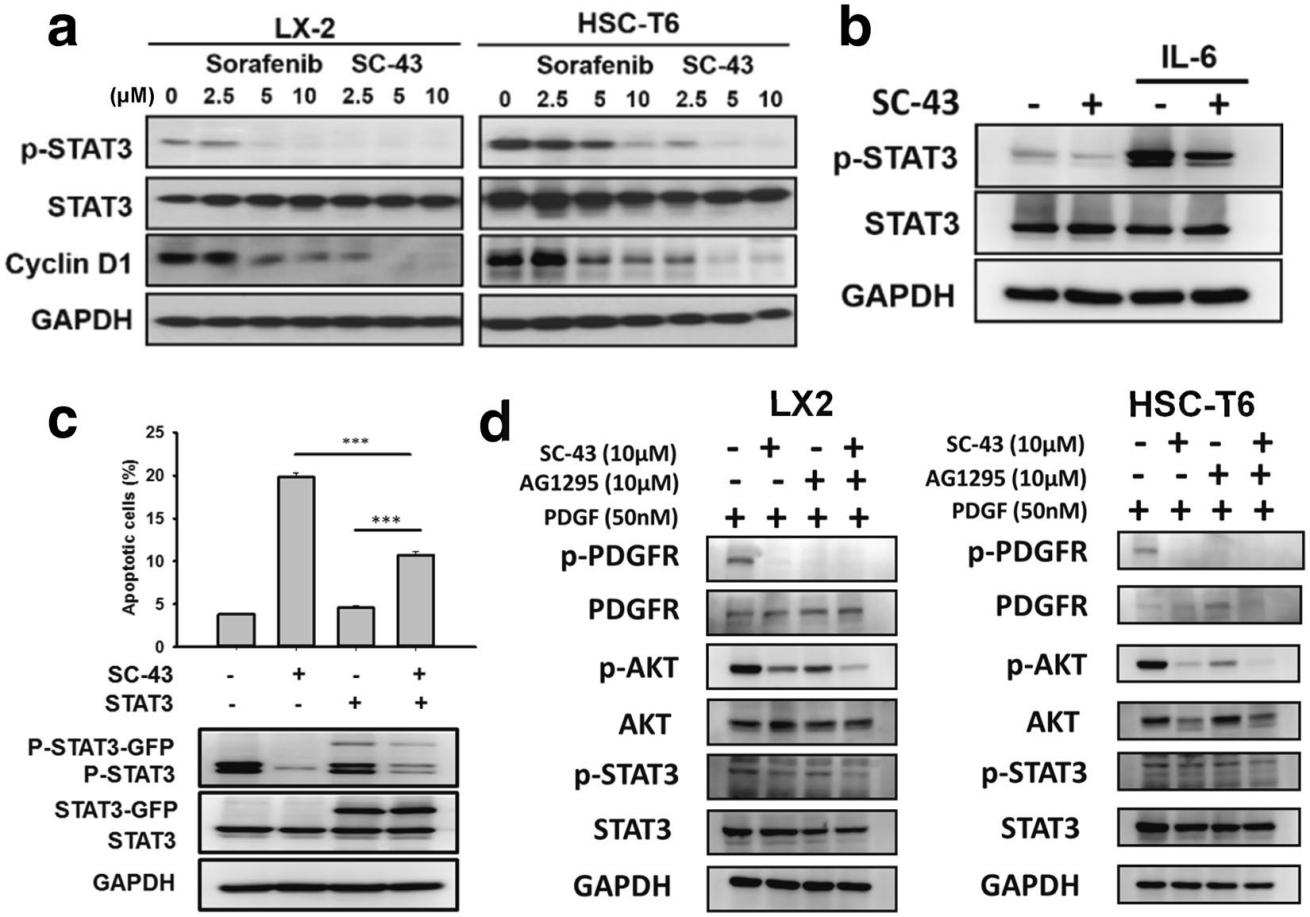
SHP-1 phosphatase plays a major role in SC-43-induced STAT3 inhibition and apoptosis. (**a**) Compared with sorafenib, the SC-43 treatment for 24 h significantly downregulated p-STAT3 and cyclin D1 dose dependently in LX2 and HSC-T6 cells. (**b**) SC-43 downregulated the IL6–STAT3 pathway. (**c**) SC-43-induced apoptosis was reverted in STAT3-overexpressed HSCs. (**d**) After administering AG-1295, a PDGFR specific inhibitor, SC-43 still downregulated p-Akt and p-STAT3.

We further investigated the effects of SC-43 on the STAT3 pathway, which is another key regulator of fibrogenesis. The SC-43 treatment showed more significant dose-escalation effects on the downregulation of p-STAT3 and cyclin D1 in LX2 and HSC-T6 cells than did sorafenib, (Fig. 6a) and in mouse primary HSCs (Supplementary Fig. S8). SC-43 also suppressed the IL-6-induced p-STAT3 upregulation (Fig. 6b). Moreover, SC-43-induced apoptosis was significantly abolished in STAT3-overexpressing HSCs (Fig. 6c). These results suggested that SC-43 more significantly induces HSC apoptosis through STAT3 pathway inhibition than does sorafenib.

In addition, after administering AG1295, a specific PDGFR inhibitor, we observed that SC-43 still downregulated p-Akt and p-STAT3, both are associated with cell proliferation and survival (Fig. 6d). These results suggested that SC-43 downregulates p-Akt and p-STAT3 through PDGFR dependent and independent mechanisms.

### SHP-1 plays a crucial role in SC-43 induced STAT3 inhibition

**Figure 7:**
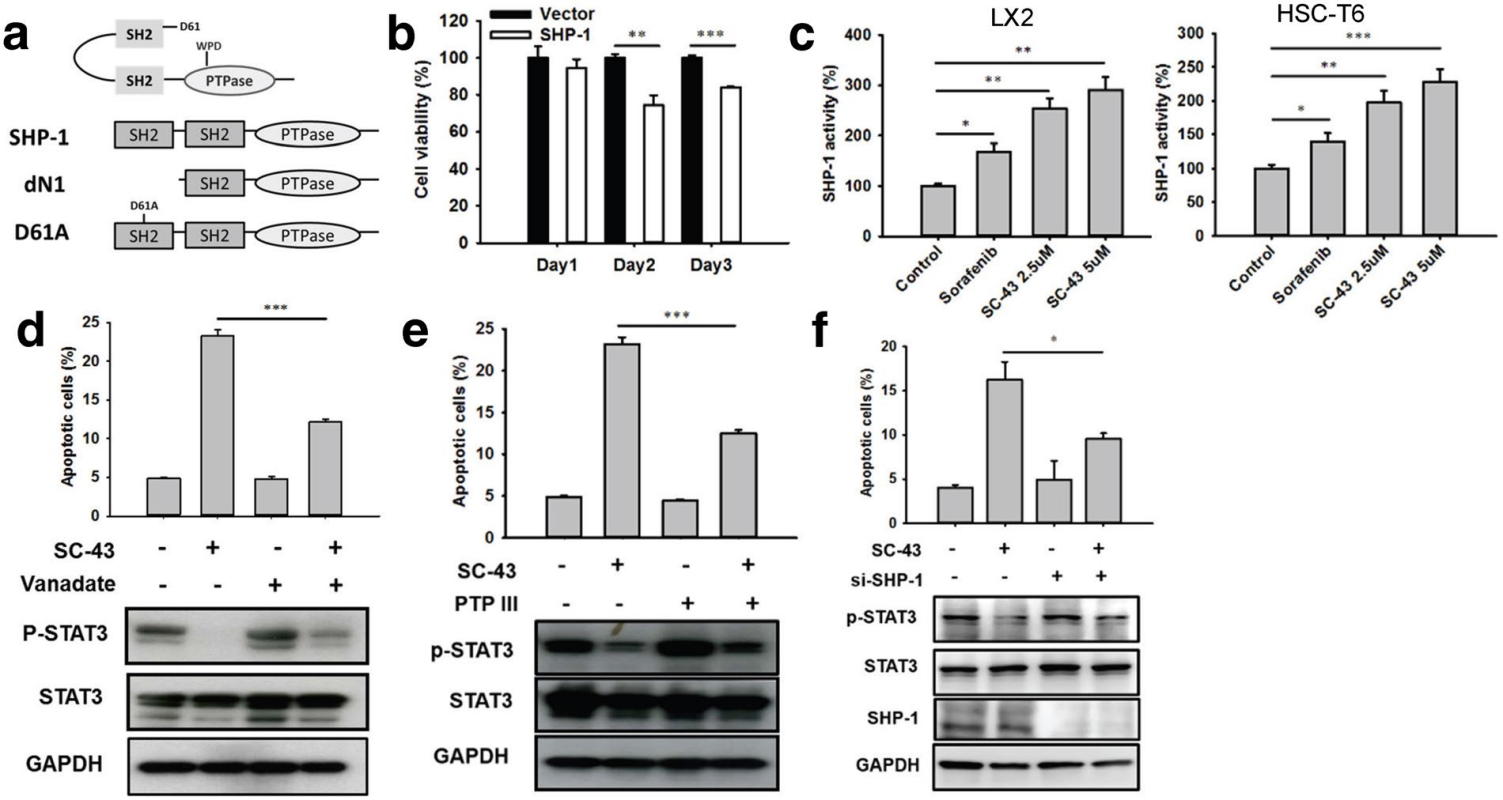
Association of SHP-1 with proliferation of HSCs. (**a**) SHP-1 and its mutants: dN1 (N-SH2 domain deletion) and D61A (single mutation of D61). (**b**) SHP-1 overexpression significantly reduced the LX2 cell viability. (**c**) The SHP-1 activity increased after sorafenib (5 µM) and SC-43 (2.5 and 5 µM) administration in both LX2 and HSC-T6 cells. (**d**) Treatment with vanadate, a nonspecific phosphatase inhibitor, upregulated p-STAT3 and reduced apoptosis. (**e**) An SHP-1-specific inhibitor (PTP inhibitor III) upregulated p-STAT3 and reduced apoptosis. (**f**) Silencing SHP-1 by using siRNA reversed the biological effects of SC-43 on p-STAT3 and apoptosis. Columns: mean and bars: standard error (n = 3–5) *P < 0.05, **P < 0.01, and ***P < 0.001.

SHP-1 contains two SH2 domains at the N-terminus (N-SH2 and C-SH2), followed by a catalytic protein tyrosine phosphatase (PTPase) domain and C-terminal tail. In its inactive form, the D61 site at the N-SH2 domain interacts with the WPD site on the PTPase domain and hinders its PTPase activity. The SHP-1 activity constitutively increased in dN1 and D61A mutants, mimicking an open conformation^10^ (Fig. 7a).

To investigate the role of SHP-1 in HSC apoptosis, we observed that SHP-1 overexpression significantly reduced cell viability since day 2 of transfection (Fig. 7b). The SC-43 treatment significantly increased the SHP-1 activity compared with sorafenib at a lower concentration both in LX2 and HSC-T6 cells (Fig. 7c). In addition, the inhibition of SHP-1 by vanadate, a nonspecific phosphatase inhibitor, rescued the SC-43-induced apoptosis of LX2 cells through p-STAT3 upregulation (Fig. 7d). Similarly, an SHP-1 specific inhibitor, PTP inhibitor III, also upregulated p-STAT3 and rescued the SC-43-induced apoptosis of LX2 cells (Fig. 7e). The antiproliferative activity of SC-43 was significantly counteracted by SHP-1 knockdown by using siRNA (Fig. 7f), suggesting that SC-43 mainly targets SHP-1, and HSC proliferation is considerably affected by SHP-1 expression and activity.

These results suggest that SHP-1 activation reduced HSC proliferation. The SC-43 treatment increased the SHP-1 activity and downregulated p-STAT3 to promote HSC apoptosis, whereas SHP-1 inhibition counteracted the effects of SC-43.

### Expression of SHP-1 mutants and proliferation of HSCs

**Figure 8:**
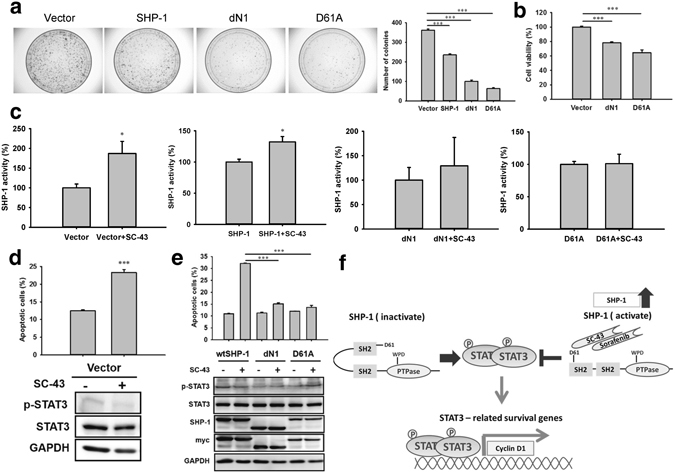
SC-43 activates SHP-1 by interaction with its inhibitory N-SH2 domain. (**a**) The ectopic expression of the SHP-1, dN1, D61A mutants significantly inhibited colony formation compared with the vector control after 14 days of incubation, as assessed using the colony formation assay. (**b**) Decreased cell viability of the dN1 and D61A mutants, as assessed using the MTS assay. (**c**) Significantly increased SHP-1 activity following the SC-43 treatment for the ectopic expression of the vector control and wild-type SHP-1 but not the dN1 and D61A mutants. The vector and myc-tagged SHP-1 mutants were expressed in LX2 cells. (**d**) Comparing the ectopic expression of the vector control, the SC-43 treatment (5 μM, 24 h) significantly increased LX2 cell apoptosis and downregulated p-STAT3. (**e**) The SC-43 treatment (5 μM, 24 h) significantly increased apoptosis and downregulated p-STAT3 in cells overexpressing wild-type SHP-1 but not the dN1 and D61A mutants. (**f**) Summary of the overall mechanisms. Columns: mean and bars: standard error (n = 3–5), *P < 0.05 and ***P < 0.001.

The associations of the ectopic expression of wild-type SHP-1, dN1, and D61A with HSC proliferation were further examined using a colony formation assay. As shown in Fig. 8a, the ectopic expression of SHP-1, dN1, and D61A significantly inhibited the number of colonies compared with the vector control. In addition, dN1 and D61A expression significantly reduced the cell viability, as observed using the MTS assay (Fig. 8b).

### SC-43 activates SHP-1 by interacting with its inhibitory N-SH2 domain

Next, we examined the effects of SC-43-induced SHP-1 activity on the ectopic expression of different SHP-1 mutants. The SC-43 treatment significantly increased the SHP-1 activity in the ectopic expression of the vector and wild-type SHP-1 but not in the ectopic expression of dN1 and D61A (Fig. 8c). Because SC-43 treatment increase the SHP-1 activity, we further investigated the phenotypic change (cell apoptosis) and p-STAT3 expression in the ectopic expression of SHP-1 mutants after SC-43 treatment. Considering the ectopic expression of the vector control and wild-type SHP-1, the SC-43 treatment significantly increased the apoptotic LX2 cells and downregulated p-STAT3 (Fig. 8d,e). However, the dN1 and D61A mutants are insensitive to SC-43 treatment considering cell apoptosis and p-STAT3 downregulation (Fig. 8e), indicating that SC-43 could not interact with the dN1 and D61A mutants to activate the SHP-1 activity.

These results suggested that the ectopic expression of SHP-1 and dN1 and D61A significantly inhibit cell proliferation. The D61 site of the inhibitory N-SH2 domain is crucial for SC-43-induced SHP-1 upregulation. The overexpression of the dN1 and D61A mutants abolished the SC-43-induced SHP-1 activation, cell apoptosis, and p-STAT3 downregulation. The antifibrotic mechanism of SC-43 in the SHP-1–STAT3 pathway is summarized in Fig. 8f.

## Discussion

The role of SHP-1 in liver fibrosis remains unclear. The SHP family contains a nonreceptor protein tyrosine phosphatase comprising two members, SHP-1 and SHP-2, which exert contrasting biological functions^11^. SHP-1 is primarily expressed in hematopoietic cells and functions as a key regulator of intracellular phosphotyrosine levels in lymphocytes^12^. SHP-1 functions as an antagonist to the growth-promoting and oncogenic potentials of tyrosine kinase; therefore, it was proposed as a tumor suppressor gene in lymphoma, leukemia, and other cancers^12^. Furthermore, SHP-1 acts as a negative regulator of several receptor tyrosine kinases, including PDGFR^13, 14^, the insulin receptor^15^, the epidermal growth factor (EGF) receptor, and vascular EGF receptor type 2^16^. Therefore, SHP-1 acts as a suppressor of the PDGFR signaling pathway and may serve as a molecular target to prevent HSC proliferation and the fibrogenic effects following HSC activation^14^. SHP-1 downregulates and subsequently blocks p-STAT3 dimerization through a direct interaction and dephosphorylation of p-STAT3^10^. By contrast, SHP-2 is a positive mediator of the Ras–extracellular signal-regulated kinase 1/2 and Akt signaling pathways^14^. In addition, SHP-1 and SHP-2 modulate cellular signals involving PI3K, Akt, JAK2, STAT, mitogen-activating protein kinases, extracellular signal-related kinases, c-Jun-amino terminal kinases, and nuclear factor-kB^11^. Overall, SHP-1 and SHP-2 modulate progenitor cell development, cellular growth, tissue inflammation, cellular chemotaxis, and cell survival^11, 16^.

In this study, we found SHP-1 inhibition by sodium stibogluconate increased fibrogenesis, suggesting SHP-1 plays important role in fibrogenesis. SHP-1 was recently reported to be abundant in the liver, with tissue specificity^17^. Moreover, we demonstrated that SHP-1 was predominately distributed in fibrotic areas. The phosphatase of these SHP-1 protein may remain in inactive states, which do not have antifibrotic effect. Only activated SHP-1 phosphatase has antifibrotic activity. Therefore, SHP-1 agonist might be used as a molecular target and tissue-specific regimen for antifibrotic therapy.

The crystal structure of the ligand-free SHP-1 protein has an autoinhibition conformation depending on the inhibitory effect of the N-SH2 domain on the catalytic PTP domain^18, 19^. The interaction between N-SH2 and PTP domains results in an intramolecular inhibition, which is stabilized by a salt bridge between Asp61 (D61) and Lys362^20^, and further blocks the entrance of phosphopeptide activators. Our results suggested that SC-43 affects SHP-1 by triggering a conformational switch, thus relieving its autoinhibition. However, the dN1 and D61A mutants lacked this autoinhibition; therefore, SC-43 could not further increase their SHP-1 activity. The docking model indicates that the trifluoromethyl group of SC-43 may dock into the pocket between the N-SH2 domain and form a hydrogen bond with Q529, which subsequently releases the active PTP domain of SHP-1^20^. The phenylcyanyl group of SC-43 is shorter than the pyridine–methylamide group of sorafenib, and the phenyl ring between urea and the phenylcyanyl moiety in SC-43 reduced its total length. These two conformational factors contribute to a more efficient fit in the N-SH2 pocket and reduce the steric-hindering effect of the N-SH2 domain, resulting in a more potent SHP-1 activation in SC-43 than in sorafenib^10^.

The antifibrotic activity of SC-43 was well demonstrated in both hepatotoxic and cholestatic animal models. In addition, compared with the control, the SC-43 treatment improved the survival of fibrotic mice. Fibrogenesis involves multiple signaling pathways. SC-43 downregulates p-Akt and p-STAT3 through PDGFR dependent and independent mechanisms might be another advantage of this compound for antifibrotic therapy. Notably, we observed cirrhosis regression after withdrawing the causative agent CCl_4_. This finding is consistent with previous studies reporting that fibrosis may reverse after withdrawing the toxic agent^21^ and similar to a recent clinical finding that sustained viral suppression by antiviral therapy can regress cirrhosis and related complications in patients with CHB^22, 23^. Even in the process of fibrosis regression, the SC-43 treatment significantly reduced the fibrosis, suggesting that HSC apoptosis during the fibrosis resolution stage might be crucial. Whether SC-43 treatment triggers other fibrinolytic mechanisms in this stage warrants further investigation. Sorafenib-induced multikinase inhibition causes various adverse reactions through off-target effects and preclude its use as an antifibrotic agent. The SC-43 lacks the Raf kinase activity, and theoretically it should have lower adverse effects. Future studies are needed to compare the *in vivo* antifibrotic activity of SC-43 with sorafenib in other animal models and investigate the adverse effects of SC-43 in preclinical studies.

In conclusion, our results suggest the relevance of the SHP-1–STAT3 pathway in fibrogenesis. SC-43 activates SHP-1 through the direct interaction of the inhibitory N-SH2 domain and promotes HSC apoptosis as the antifibrotic mechanism. Furthermore, SC-43 reduces liver fibrosis in both hepatotoxic and cholestatic fibrosis mouse models, indicating that the SHP-1 agonist is a potential target for antifibrotic drug discovery.

## Methods

### Materials

SC-43, or 1-(4-chloro-3-(trifluoromethyl)phenyl)-3-(3-(4-cyanophenoxy)phenyl) urea, was synthesized by Dr. Chung-Wai Shiau at National Yang-Ming University. Sorafenib (Nexavar) was kindly provided by Bayer HealthCare AG (Berlin, Germany). Antibodies for alpha-smooth muscle actin (α-SMA), P-STAT3 (Tyr705), STAT3, cyclin D1, glyceraldehyde-3-phosphate dehydrogenase, P-Smad2 (Ser465/467), P-Smad3 (Ser423/425), Smad2, Smad3, poly (ADP-ribose) polymerase (PARP), platelet-derived growth factor receptor (PDGFR)-β, P-PDGFR-β (Tyr857), and P-Akt (Ser473) were purchased from Cell Signaling (Danvers, MA, USA). Akt was purchased from Santa Cruz Biotechnology (San Diego, CA, USA). Furthermore, sodium vanadate was purchased from Cayman Chemical (Ann Arbor, MI, USA). PTP inhibitor III was purchased from Calbiochem (San Diego, CA, USA). Cremaphor and sodium stibogluconate were obtained from Sigma (Saint Louis, MO, USA).

### SHP-1 expression in patients with liver fibrosis

We enrolled 25 patients with CHB with different degrees of liver fibrosis (n = 5 in Metavir fibrosis score F0, F1, F2, F3 and F4, respectively). Their biopsied liver tissues were stained with SHP-1. The study conformed to the ethical guidelines of the 1975 Declaration of Helsinki and was approved by the Institutional Review Board of the National Taiwan University Hospital. Written informed consent was obtained from all patients at enrollment.

### Liver fibrosis mouse model

Eight-week-old male C57BL/6JNarl mice or Balb/C mice (20–25 g) were obtained from the National Laboratory Animal Center, Taiwan. In the hepatotoxic model, CCl_4_ (1 µL/g) was administered through a biweekly intraperitoneal injection to the C57BL/6JNarl mice for 4 or 8 weeks. A vehicle (cremaphor), SC-43 (5, 10, or 20 mg/kg), or sorafenib (10 mg/kg) were administered through oral gavage for 5 days a week during a specified schedule until sacrifice. The concentrations, route and frequency of SC-43 and sorafenib were selected according to previous published results^10^. Sorafenib served as the positive control because of its proven antifibrotic activity^9^.

In the BDL model, the common bile duct was double ligated, followed by resection in the Balb/C mice. A vehicle or SC-43 were administered through oral gavage daily from day 1 or 8 until sacrifice on day 14.

Sodium stibogluconate, a specific SHP-1 inhibitor^24^, were administered daily (20 mg/kg/day by intraperitoneal injection) in the CCl_4_ model for 2 weeks and in the BDL model for 1 week to investigate the effect of SHP-1 inhibition on fibrogenesis. All experimental animal procedures were in accordance with protocols approved by the Institutional Laboratory Animal Care and Use Committee of National Taiwan University.

### Histological analysis of liver fibrosis

Mouse liver specimens were preserved in 10% formaldehyde, dehydrated in a graded alcohol series, embedded in paraffin blocks, sectioned to 3-μm thickness, placed on glass slides, and stained using a Picrosirius Red Stain kit (ScyTek, Logan, UT, USA), according to the manufacturer’s instructions. The liver fibrosis severity was graded according to the Ishak fibrosis scoring system. The collagen-positive area was evaluated through densitometry by using ImageJ software. The liver hydroxyproline concentration was measured using a hydroxyproline assay kit (Biovision, Milpitas, CA, USA), according to the manufacturer’s instructions. Immunohistochemical staining was performed using the Leica BOND-MAX autostainer (Leica Biosystems), according to the manufacturer’s protocol. The TUNEL assay (Roche diagnostics, Mannheim, Germany) was performed according to the instructions.

### Hepatic stellate cell culture

HSC-T6 and LX2 cells were immortalized rat and human HSC lines, respectively, and were kindly provided by Prof. Scott Friedman from Mount Sinai Hospital, New York. Primary mouse HSCs were isolated according to previously described procedures^9^. HSC-T6 cells were cultured in Waymouth medium and LX2 cells and primary mouse HSCs in Dulbecco’s modified Eagle medium (DMEM; Invitrogen, Carlsbad, CA, USA). All media were supplemented with 10% fetal bovine serum (FBS), 100 units/mL penicillin G, 100 μg/mL streptomycin sulfate, and 2 mM L-glutamine. All cells were cultured in a 37 °C humidified incubator under 5% CO_2_ in air. The quiescent HSCs were activated following FBS supplementation. For *in vitro* studies, various concentrations of SC-43 and sorafenib were dissolved in dimethyl sulfoxide and subsequently added to the cells in 5% FBS for the scheduled incubation duration.

### Cell viability and apoptosis analysis

HSC-T6 or LX2 cells or primary mouse HSCs were seeded in 96-well, flat-bottomed plates with 5000 cells per well with 10% FBS for 24 h. The cells were exposed to various concentrations of SC-43 or sorafenib for 24 h and 48 h. The effects of the individual agents on cell viability were evaluated using a CellTiter 96 AQueous one solution cell proliferation assay containing 3-(4,5-dimethylthiazol-2-yl)-5-(3-carboxymethoxyphenyl)-2-(4-sulfophenyl)-2H-tetrazolium (MTS), an inner salt (Promega, Madison, WI, USA) in triplicate, according to the manufacturer’s protocol. After SC-43 or sorafenib treatment for 24 h, the percentage of apoptotic cells were determined through Annexin V and propidium iodide (PI) double staining after pooling both early (Annexin V+/PI−) and late (Annexin V+/PI+) apoptotic cells on a BD FACS Verse flow cytometer (BD, Franklin Lakes, NJ, USA). The cell cycles were evaluated by PI staining.

### Western blotting

Whole cell extracts were obtained using a radioimmunoprecipitation assay (RIPA) buffer (Merck Millipore, Temecula CA, USA), and the protein concentrations were quantified using a BCA protein assay kit (Thermo, Rockford, IL, USA). Twenty-five micrograms of a sample protein was loaded onto sodium dodecyl sulfate–acrylamide gels of various percentages, electrophoresed, and transferred to polyvinylidene fluoride membranes. After treatment with 5% bovine serum albumin in Tris-buffered saline and Tween 20 for 1 h, the membranes were subsequently incubated overnight with the appropriate primary antibodies. Following extensive washing, the membranes were incubated for 1 h with a blocking buffer containing horseradish peroxidase (HRP)-conjugated secondary antibodies. The proteins were detected using the Immobilon Western Chemiluminescent HRP substrate (Millipore, Billerica, MA, USA) or the ECL detection system (UVP, LLC, Upland, CA, USA).

### Transforming growth factor -β induction

For transforming growth factor (TGF)-β induction, the HSCs were serum deprived for 4 h, subsequently treated with 10 μM SC-43 for 4 h, and followed by stimulation with 10 ng/mL recombinant human TGF-β1 (R & D Systems, Minneapolis, MN, USA) for 20 min.

### Platelet-derived growth factor BB induction

For PDGF-BB induction, the HSCs were serum deprived for 4 h, treated with 10 μM SC-43 for 4 h, followed by induction with 50 ng/mL recombinant human or rat PDGF-BB (R&D Systems) for 10 min on LX2 or HSC-T6 cells, respectively.

### Interleukin-6 stimulation

For interleukin (IL)-6 stimulation, the HSCs were serum deprived for 4 h, treated with 10 μM SC-43 for 4 h, followed by induction with 100 ng/mL IL-6 (R&D Systems) for 30 min.

### Plasmid, siRNA, and transfection

Rat STAT3 (Open Biosystem, Pittsburgh PA, USA) was constructed into a pLVX–AcGFP-N1 expression vector (Clontech, Mountain View, CA, USA), which was later cotransfected into 293FT cells in addition to lentiviral packaging and expression vectors (P8.91 and VSV-G) by using the Lipofectamine 2000 transfection reagent (Invitrogen). The lentiviral supernatant was harvested 48 h post transfection and used to infect 5 × 10^5^ HSC-T6 cells, which were seeded on a 6-cm dish; the rat STAT3 stable overexpression clone was generated.

Plasmids encoding the human wild-type SHP-1 and SHP-1 mutant, in which the N-SH2 domain was truncated (dN1) or one aspartic acid at site 61 was changed into an alanine residue (D61A) were cloned into the pCMV6-entry vector with myc-tag. These mutants were confirmed through DNA sequencing. Smart-pool siRNA, including the control (D-001810-10), and SHP-1 (PTPN6, L-009778-00-0005) were obtained from Dharmacon Inc (Chicago, IL, USA). For transient expression, SHP-1 plasmids or siRNA (final concentration, 100 nM) with the Lipofectamine 2000 transfection reagent were pretransfected into the LX2 cells for 24 h, according to the manufacturer’s instructions.

### SHP-1 phosphatase activity

After 24 h of SC43 treatment with 5% FBS in DMEM in HSCs, protein extracts were incubated overnight with an anti-SHP-1 antibody in the RIPA lysis buffer. Protein G-Sepharose 4 Fast Flow (GE Healthcare BioScience, Piscataway, NJ, USA) was added into each sample, followed by incubation for 3 h at 4 °C, with rotation. The RediPlate 96 EnzChek Tyrosine Phosphatase Assay Kit (R-22067, Molecular Probes, Carlsbad, CA, USA) was used for determining the SHP-1 activity, according to the manufacturer’s protocol.

### Colony formation assay

LX2 cells were plated in 10-cm dishes (1500–5000 cells per dish) and cultured in DMEM for 2 weeks. The cells were subsequently fixed with 4% formaldehyde and stained with 0.1% crystal violet.

### Statistical analysis

Continuous variables are presented as mean (standard error), and categorical data are presented as number (percentage), as appropriate. Differences between subgroups were evaluated using the Student t test. Spearman’s correlation was used for the association between SHP-1 and aspartate aminotransferase (AST) to platelet ratio index (APRI, [AST/AST upper limit of normal]/platelet x 100), a biomarker for liver fibrosis^25^, or serum alanine aminotransferase (ALT). The mouse survival rate was estimated using the Kaplan–Meier method. The log-rank test was used to determine the statistical differences in survival of the different experimental groups. Statistical analysis was performed using STATA (version 13, Stata Corp, College Station, TX, USA). All tests were two-sided, and P < 0.05 was considered significant.

## Electronic supplementary material

Supplementary Data
