# Supplementary material for: Src-homology protein tyrosine phosphatase-1 agonist, SC-43, reduces liver fibrosis
Source: Sci Rep. 2017 May 11;7:1728. doi: 10.1038/s41598-017-01572-z (PMC5431996; doi:10.1038/s41598-017-01572-z)

Supplementary Data

Title: Src-homology protein tyrosine phosphatase-1 agonist, SC-43, reduces liver fibrosis

Authors:

Tung-Hung Su, Chung-Wai Shiau, Ping Jao, Nian-Jie Yang, Wei-Tien Tai, Chun-Jen Liu, Tai-Chung Tseng, Hung-Chih Yang, Chen-Hua Liu, Kai-Wen Huang, Ting-Chen Hu, Yu-Jen Huang, Yao-Ming Wu, Li-Ju Chen, Pei-Jer Chen, Ding-Shinn Chen, Kuen-Feng Chen, Jia-Horng Kao

Figure S1. The positive correlation between SHP-1 expression and ALT.

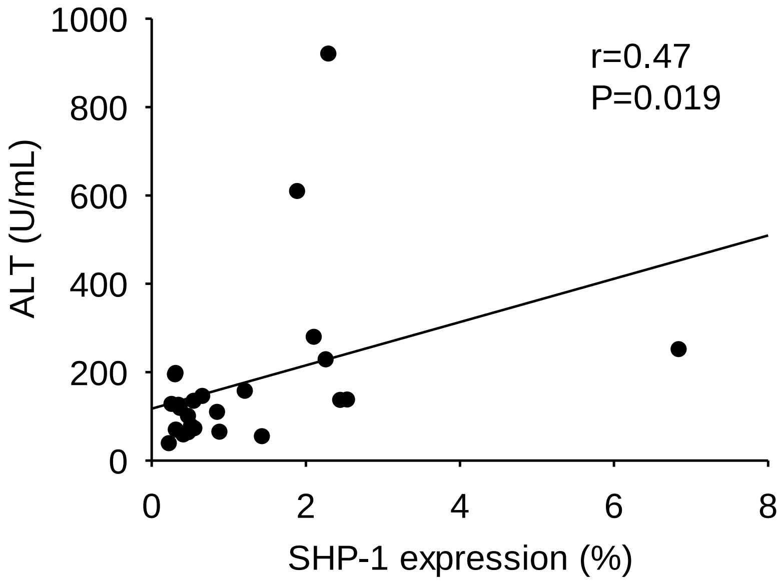

Figure S2. SHP-1 inhibition increased liver fibrosis in CCl_4-induced fibrosis mouse model.

Sodium stibogluconate, a specific SHP-1 inhibitor, was administered during CCl_4 induction for 2 weeks. Representative images after sirius red staining and collagen-positive area quantification. Scale bar: 100 μm. Columns: mean and bars: standard error. *P < 0.05 compared with the vehicle group. N = 9 in each group.

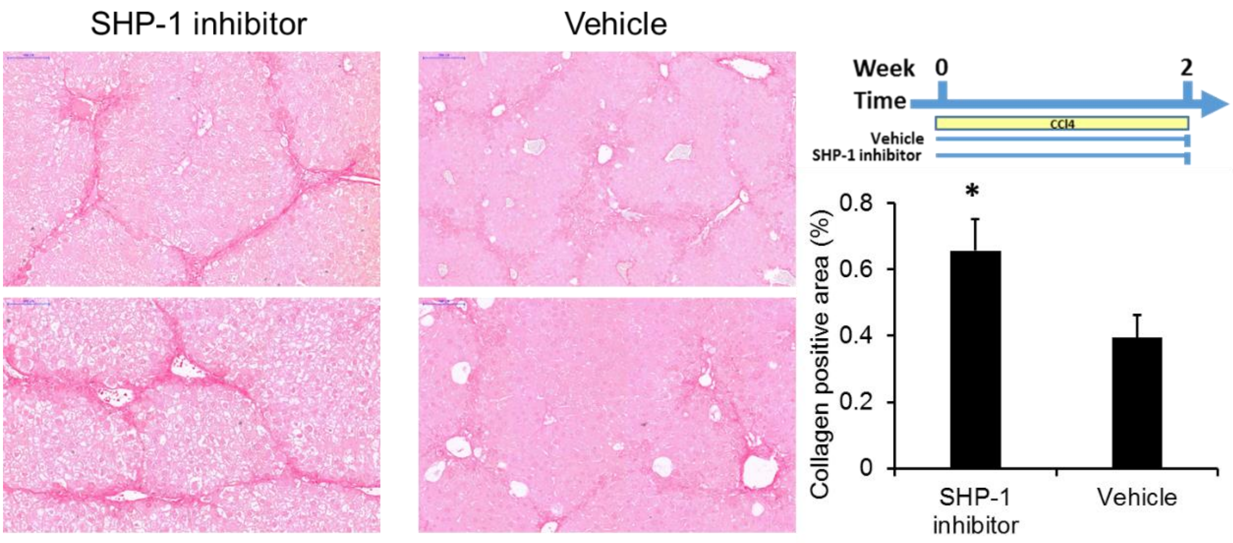

Figure S3. SHP-1 inhibition increased liver fibrosis in bile duct ligation fibrosis mouse model.

Sodium stibogluconate, a specific SHP-1 inhibitor, was administered after BDL for 7 days. Representative images after sirius red staining and collagen-positive area quantification. Scale bar: 100 μm. Columns: mean and bars: standard error. N =8 in each group.

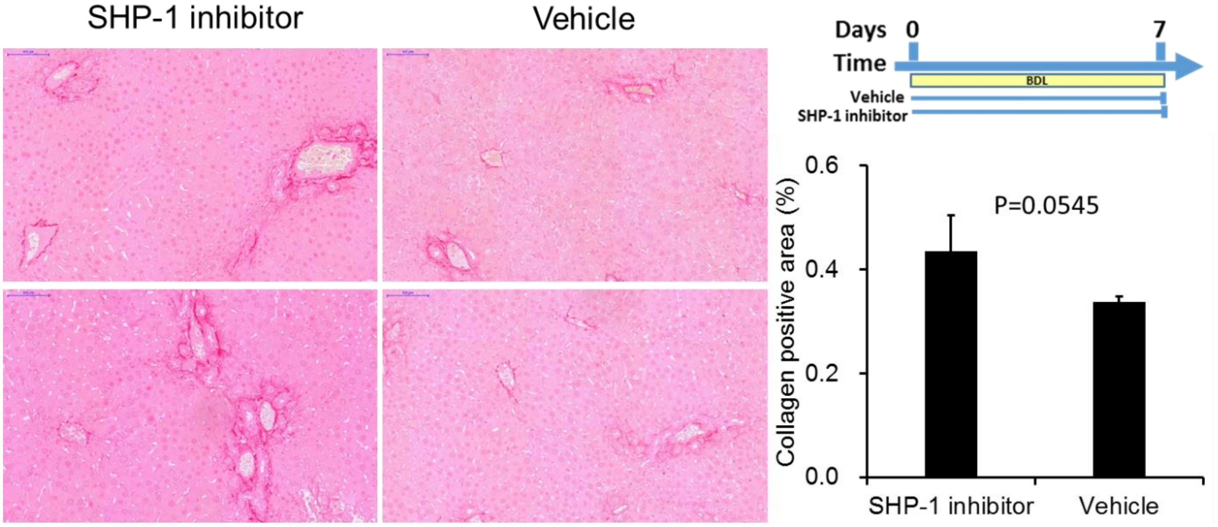

Figure S4. SC-43 treatment reduces the α-SMA expression in CCl_4-induced liver fibrosis mouse model.

Representative images after α-SMA staining and quantification. Scale bar: 100 μm. Columns: mean and bars: standard error. N=3 in each group.

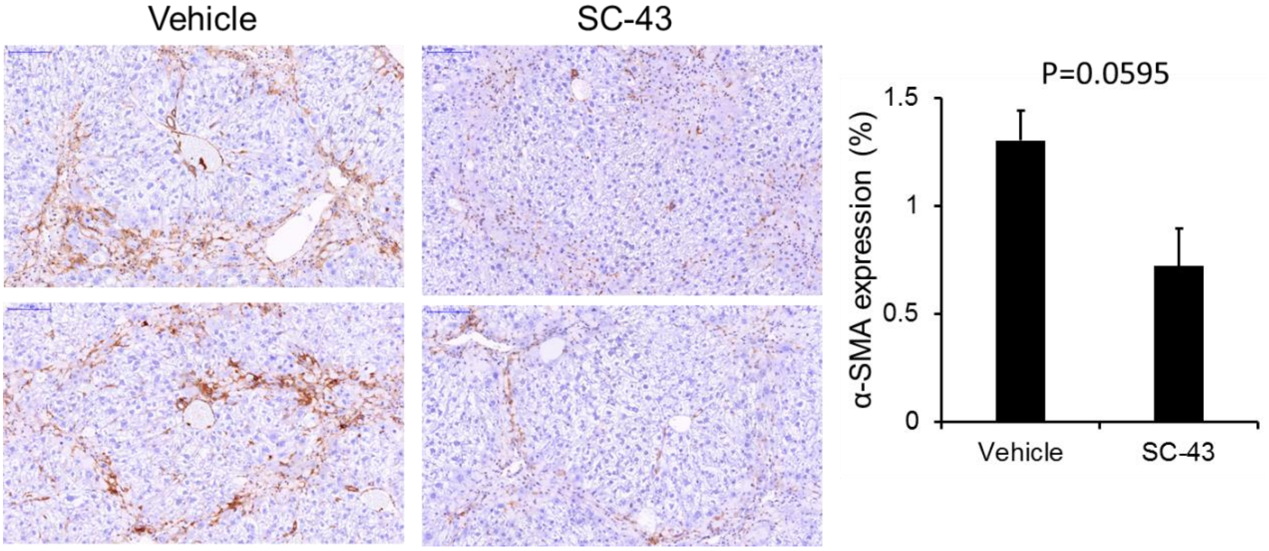

Figure S5. SC-43 treatment increases apoptotic hepatic stellate cells in CCl4-induced liver fibrosis mouse model.

Representative images after TUNEL staining for apoptotic cells and quantification. Scale bar: 100 μm. Columns: mean and bars: standard error. *P < 0.05 compared with the vehicle group. N=4-6 in each group.

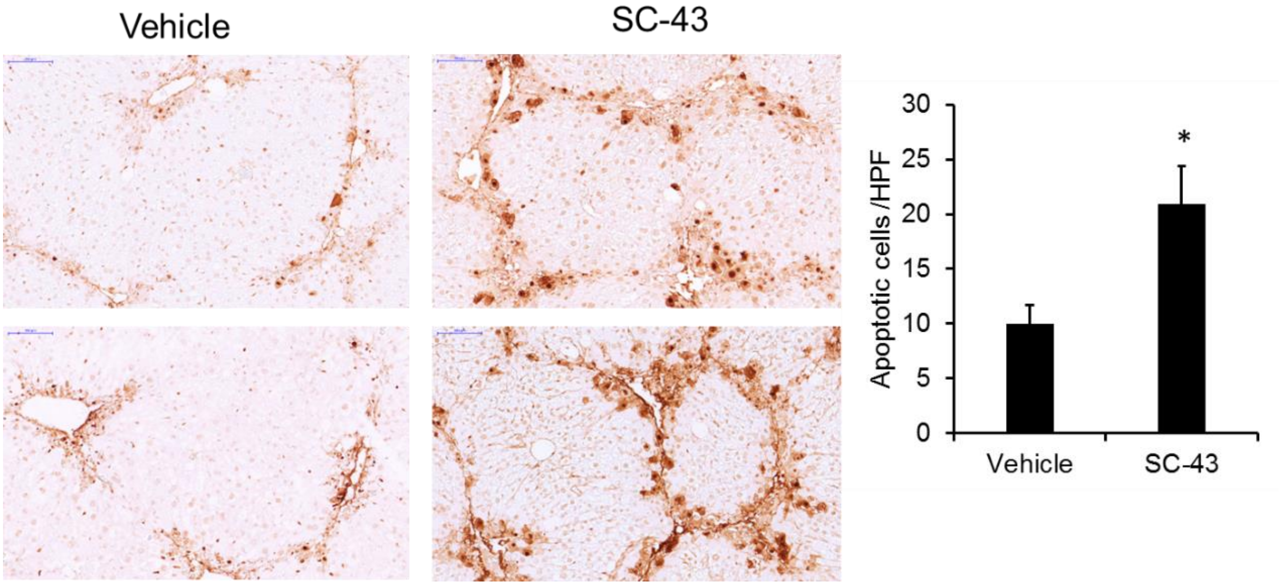

Figure S6. The liver hydroxyproline level in bile duct ligation mouse models.

(a) In the BDL fibrosis prevention model. (b) In the BDL fibrosis therapy model.

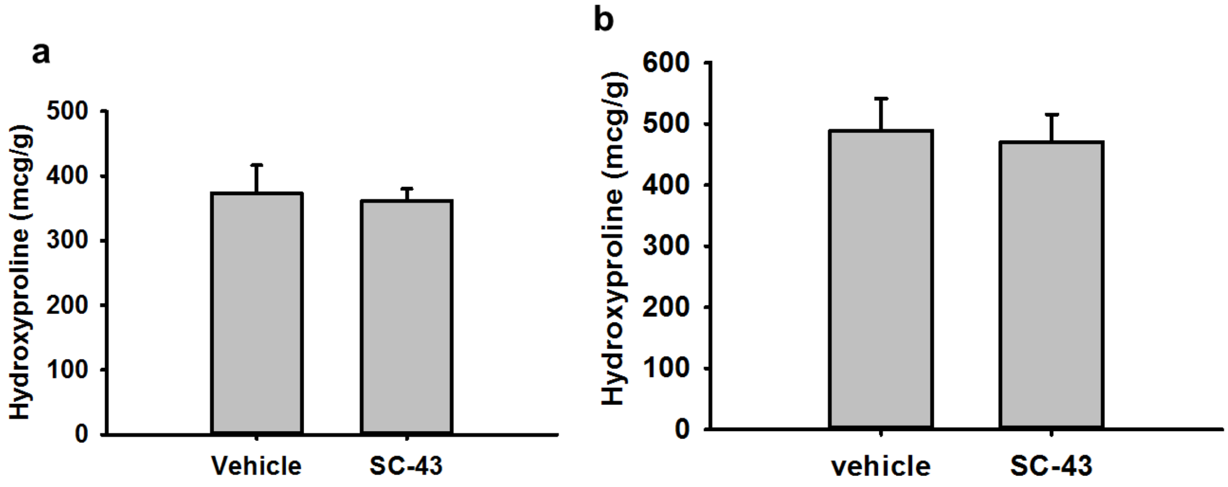

Figure S7. The cell cycle is HSCs after SC-43 treatment.

(a) Representative image of cell cycles in LX2 cells. (b) The increased Sub-G1 phase on LX2 and HSC-T6 cells after SC-43 treatment.

(a)

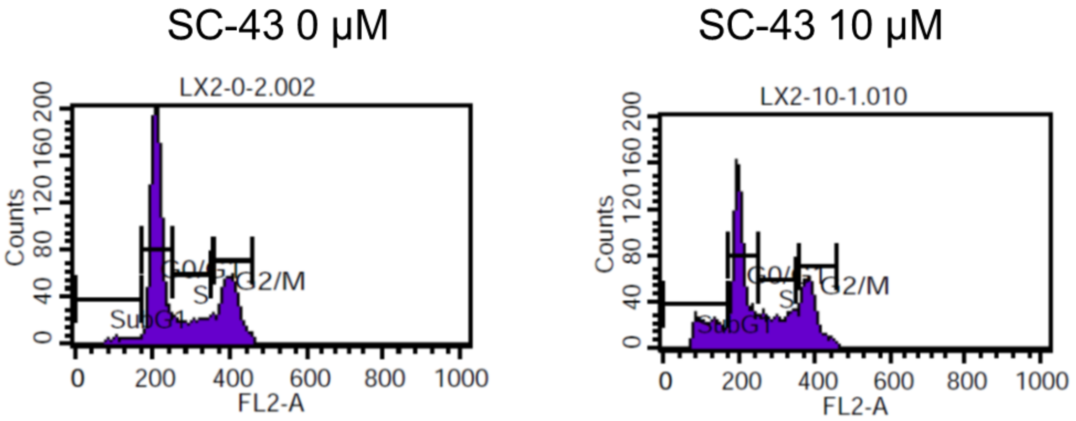

(b)

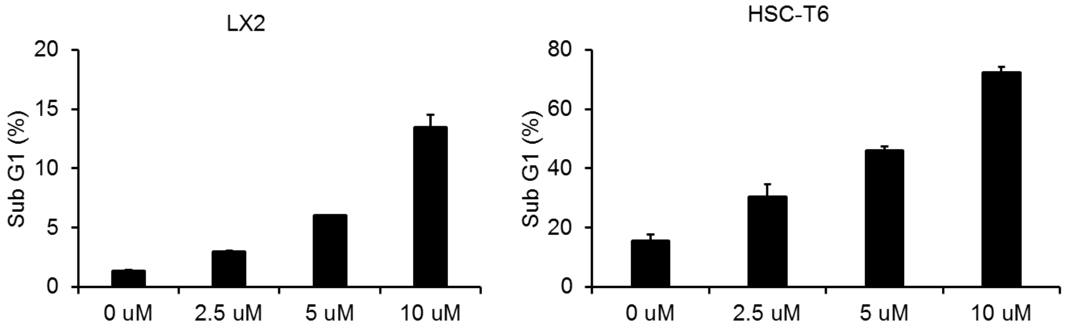

Figure S8. SC-43 treatment downregulates phospho-STAT3 in primary mouse hepatic stellate cells

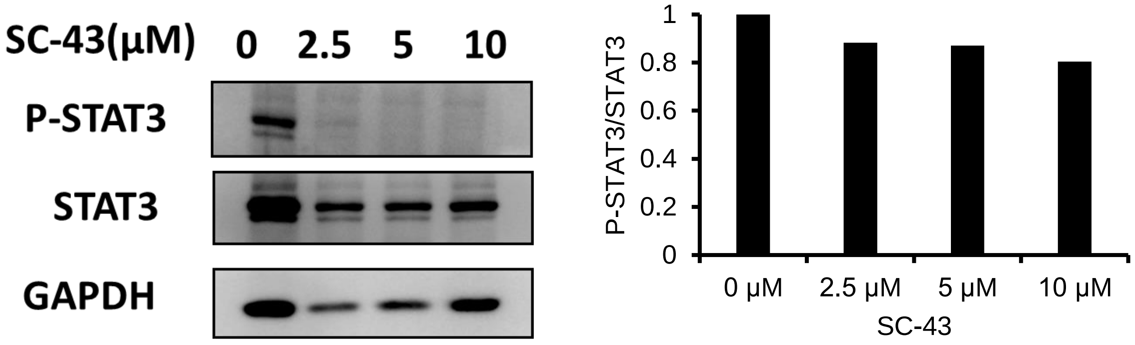

Supplement: Supplementary file 1 — Supplementary Data [file 41598_2017_1572_MOESM1_ESM.pdf]
